# Novel mutations in *RDH5* cause fundus albipunctatus in two consanguineous Pakistani families

**Published:** 2012-06-13

**Authors:** Muhammad Ajmal, Muhammad Imran Khan, Kornelia Neveling, Yar Muhammad Khan, Syeda Hafiza Benish Ali, Waqas Ahmed, Muhammad Safdar Iqbal, Maleeha Azam, Anneke I. den Hollander, Rob W.J. Collin, Raheel Qamar, Frans P.M. Cremers

**Affiliations:** 1 Department of Biosciences, Faculty of Science, COMSATS Institute of Information Technology, Islamabad, Pakistan; 2 Department of Human Genetics, Radboud University Nijmegen Medical Centre, Nijmegen, The Netherlands; 3 Shifa College of Medicine, Islamabad, Pakistan; 4 Institute for Genetic and Metabolic Disorders, Radboud University Nijmegen Medical Center, Nijmegen, The Netherlands; 5 Department of Chemistry, University of Science and Technology, Bannu-28100, Pakistan; 6 Department of Ophthalmology, Nishtar Hospital, Multan, Pakistan; 7 Department of Ophthalmology, Radboud University Nijmegen Medical Centre, Nijmegen, The Netherlands; 8 Nijmegen Centre for Molecular Life Sciences, Radboud University Nijmegen Medical Centre, Nijmegen, The Netherlands

## Abstract

**Purpose:**

To identify the underlying genetic causes of fundus albipunctatus (FA), a rare form of congenital stationary night blindness that is characterized by the presence of white dots in the midperiphery of the retina and delayed dark adaptation, in Pakistan.

**Methods:**

Two families with FA were identified by fundus examination, and genome-wide single nucleotide polymorphism genotyping was performed for two individuals from family A and six individuals from family B. Genotyping data were subsequently used to identify the identical homozygous regions present in the affected individuals of both families using the online homozygosity mapping tool Homozygosity Mapper. Candidate genes selected from the homozygous regions were sequenced.

**Results:**

Three identical homozygous regions were identified in affected persons of family A (on chromosomes 8, 10, and 12), whereas a single shared homozygous region on chromosome 12 was found in family B. In both families, the homozygous region on chromosome 12 harbored the retinol dehydrogenase 5 (*RDH5*) gene, in which mutations are known to be causative of FA. *RDH5* sequence analysis revealed a novel five base pair deletion, c.913_917delGTGCT (p.Val305Hisfs*29), in family A, and a novel missense mutation, c.758T>G (p.Met253Arg), in family B.

**Conclusions:**

We identified two novel disease-causing *RDH5* mutations in Pakistani families with FA, which will improve diagnosis and genetic counseling, and may even lead to treatment of this disease in these families.

## Introduction

Fundus albipunctatus (FA; OMIM:136880), or flecked retina disease, was described for the first time by Lauber [1]. FA is a rare form of congenital stationary night blindness and is characterized by the presence of typical white dots on the whole fundus or concentrated in the midperipheral region of the retina, with or without macular involvement, and a delay in dark adaptation. The inheritance pattern of FA is autosomal recessive [2-5]. In one family, a male and his two daughters showed FA, which could be due to autosomal dominant or pseudodominant (i.e., autosomal recessive) inheritance [6]. Mutations in three genes–retinol dehydrogenase 5 (*RDH5*), retinaldehyde-binding protein 1 (*RLBP1*), and retinal pigment epithelium–specific protein (*RPE65*)–are known to be associated with FA [7-10]. Retinitis punctata albescens has similar phenotypic characteristics but is progressive in nature and is mostly caused by mutations in *RLBP1* [8].

FA-causing mutations were first identified in *RDH5*, which is expressed predominantly in the retinal pigment epithelium (RPE) [7]. *RDH5* encodes an enzyme that is part of the visual cycle, which involves a series of specialized enzymes and retinoid binding proteins that are essential for the regeneration of the 11-*cis* retinal chromophore [11-14]. RDH5 consists of 318 amino acids and is highly conserved among different species [15]. Within the RPE cells, RDH5 resides in the smooth endoplasmic reticulum [16] where it is principally involved in chromophore regeneration by catalyzing the final step in the biosynthesis of 11-*cis* retinal [7,17-20].

The current study explores the molecular mechanisms behind FA in Pakistani families, using high-density single nucleotide polymorphism (SNP) microarrays and sequence analysis of known FA genes located in the identified homozygous regions. Using this approach, we identified two novel mutations in *RDH5* in two families with FA.

## Methods

### Approval of the study

Approval for this study was granted by the Ethics Committee/Institutional Review Board of Shifa College of Medicine/Shifa International Hospital, Islamabad. Signed informed consent was obtained from members of both families participating in the current study.

### Family collection and clinical evaluation

**Figure 1:**
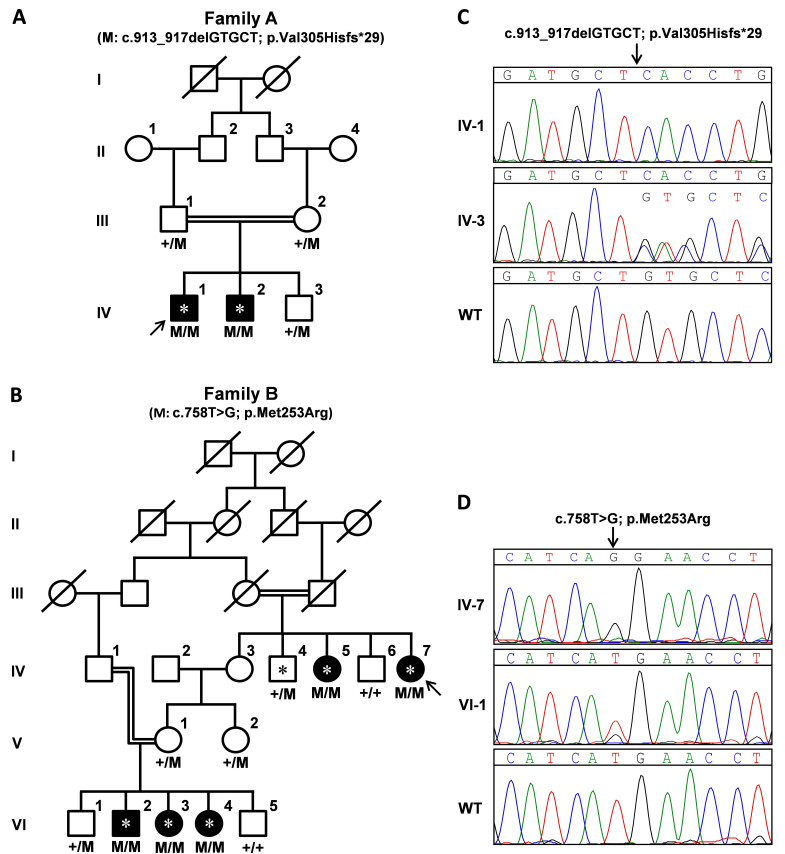
Pedigrees and sequencing results. **A**: Segregation of the mutation in family A. **B**: Segregation of the mutation in family B. **C** and **D**: Sequence electropherograms of affected individuals carrying homozygous variants (upper panels) and unaffected heterozygous carriers (middle panels) of families A (**C**) and B (**D**), along with the results of a control individual (wild-type [wt], lower panels). Arrows point to the probands; individuals tested with single nucleotide polymorphism (SNP) microarrays are indicated with asterisks.

Families A and B (Figure 1) reside in remote areas of Pakistan and were part of a cohort of 83 families with retinitis pigmentosa and associated retinal diseases. Blood samples were collected from affected and normal individuals of both families and DNA was extracted by a standard protocol [21]. Pedigrees were drawn using Haplopainter [22]. Both families were clinically evaluated by fundus examination; in addition, electroretinography (ERG) measurements were recorded for family A.

### Homozygosity mapping analysis

All affected individuals from both families and one healthy person from family B were subjected to high-density HumanOmniExpress (>700 K; Illumina Inc., San Diego, CA) single nucleotide polymorphism (SNP) microarray analysis. Genotyping data were analyzed with the online tool Homozygosity Mapper [23]. Haplotypes of affected and normal individuals were compared in each family to identify the identical homozygous regions shared by all affected individuals.

### Primer design and *RDH5* sequence analysis

The online tool Primer3 [24] was used to design PCR primers (Table 1). The five exons of *RDH5*, including their flanking exon-intron boundaries, were amplified by PCR using standard conditions and reagents. PCR-amplified exonic fragments were electrophoretically separated on 2% agarose gels containing ethidium bromide and DNA bands were visualized under ultraviolet transillumination. PCR clean-up purification plates (NucleoFast® 96 PCR; Cat. No. 743100.10, Macherey-Nagel, Düren, Germany) were used to purify the amplified fragments according to the manufacturer’s protocol. Briefly, 20 µl of each amplified PCR product was transferred to Nucleofast 96 PCR plate. Wells were filled up to 100 µl volume with RNase-free water to ensure the uniform loading. Contaminants were removed by ultrafilteration with the help of a vacuum apparatus for 10 min. Thirty µl of RNase-free water was poured in each well and DNA was recovered by thorough mixing with a multi-channel pipette. Sanger sequencing was then performed with Big Dye Terminator version 3 and analyzed on a 3730 DNA analyzer (Applied Biosystems, Inc., Foster City, CA).

**Table 1:** Primer sequences of *RDH5*.

**Exon**	**Forward primer (5′-3′)**	**Reverse primer (5′-3′)**	**Amplified fragment length (bp)**
1	CTAGGCAAATCTGGCCTCTG	GGTCCACCTCAGAGTTGTGG	396
2	GGAAAGGGCTTGAGGGC	GACTGTGGGGATCAGGACAC	450
3	CTCCCAGGAAGAAGAGGGAG	CACCTCTGCTGGCCCAC	399
4	ATGTCCCTCAAAGTCCCCTC	AGGCTTATGCAGGACTGGC	301
5	GGCCCCAGAAGACAGTACC	CGTGCAGCTGTAGATGTGAG	589

Vector NTI Advance (TM) 2011 software from Invitrogen Corporation (Carlsbad, CA) was used to analyze the sequencing results of *RDH5* exons.

### In silico analysis

Sorting Intolerant from Tolerant (SIFT), Polymorphism Phenotyping v2 (Polyphen-2), and Mutation Taster [25] were used to assess the possible pathological nature of the missense variant identified in this study. Project HOPE [26] was used to analyze and predict the structural variations in mutant RDH5.

### Amino acid conservation

RDH5 protein sequences from different species including human (*H. sapiens*, ENSP00000257895), macaque (*M. mulatta*, ENSMMUP00000017380), mouse (*M. musculus*, ENSMUSP00000026406), dog (*C. familiaris*, ENSCAFP00000000084), cow (*B. taurus*, ENSBTAP00000056512), cat (*F. catus*, ENSFCAP00000012945), tetraodon (*T. nigroviridis*, ENSTNIP00000022889), and round worm (*C. elegans*, F35B12.2) were aligned using Vector NTI Advance™ 2011 to check the evolutionary conservation of the substituted amino acid in RDH5.

## Results

### Clinical studies

**Figure 2:**
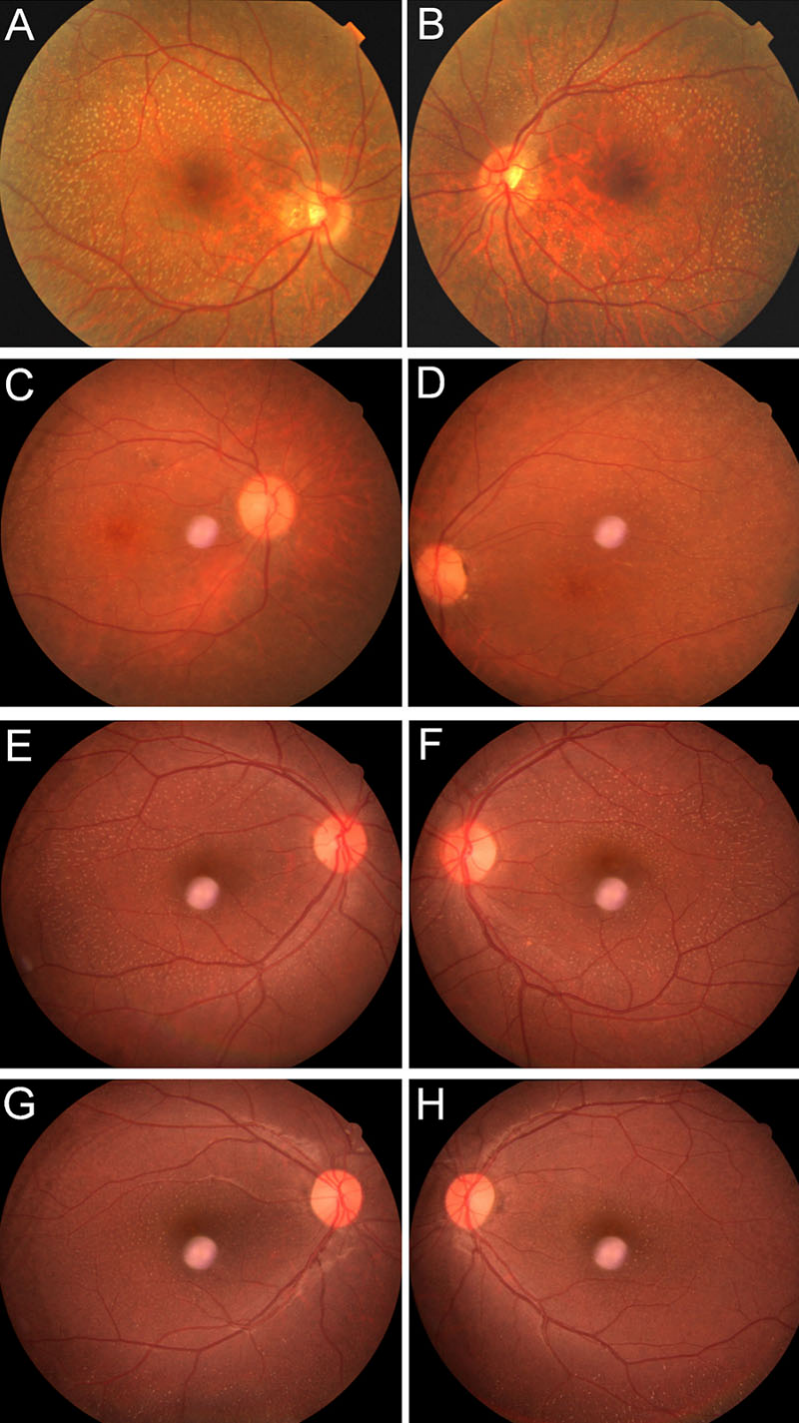
Fundus photographs of affected individuals from both families. **A**, **B**: Right and left eye, respectively, of affected individual IV-1 of family A (see arrow, Figure 1A). **C**, **D**: Right and left eye, respectively, of affected individual IV-7 of family B (see arrow, Figure 1B). **E**, **F**: Right and left eye, respectively, of affected individual VI-2 of family B. **G**, **H**: Right and left eye, respectively, of affected individual VI-3 of family B.

Initial symptoms of visual complaints in patients from both families were observed from early childhood. Fundus examination of affected individuals revealed the presence of white dots typical of FA in the midperiphery of the retina (Figure 2; Table 2). ERG responses of cone and rod photoreceptors were diminished in affected individual IV-1 of family A (Table 3). This individual had daytime vision problems, which confirms that cone photoreceptors were also affected. Macular degeneration was also observed in individual IV-1 of family A and individual IV-7 of family B. ERG results were not available for family B. The visual acuity (VA) of affected individual IV-7 of family B was different from the VAs of other individuals (VI-2, VI-3) of this family, and the density of white dots was also variable, which indicates intrafamilial phenotypic variability. Affected individuals of family B had normal daytime vision.

**Table 2:** Clinical features of affected individuals in both families.

**Individual**	**Age (years)**	**VA (RE, LE)**	**Fundus phenotype**	**RPE degeneration**	**Retinoscopy**
Family A, IV-1	35	6/12, 6/12	White dots, macular degenerative changes	Yes	Not determined
Family B, IV-7	45	6/18, 6/12	White dots, macular degenerative changes	Yes	Hypermetropia
Family B, VI-2	17	6/6, 6/6	White dots, macula healthy	No	Low hypermetropia
Family B, VI-3	10	6/6, 6/6	White dots, macula healthy	No	Low hypermetropia

LE, left eye; RE, right eye; RPE, retinal pigment epithilium; VA, visual acuity.

**Table 3:** ERG responses of affected individual IV-1 of Family A in comparison with ERG responses of a control individual.

**Measured parameters using monopolar electrodes**	**Adaptation**	**Flash strength** **(cd•s/m^2^)**	**Proband family A**	**Control**	**Normal values (Age=40 years)**
Scotopic 25 dB b-wave amplitude (µV)	Dark	0.01	45.1	173.20	>141
Scotopic 0 dB b-wave amplitude (µV)	Dark	3.0	149.1	496.80	>387
Oscillatory potential amplitude (µV)	Dark	3.0	80.3	123.90	>75
Photopic 0 dB b-wave amplitude (µV)	Light	3.0	70.7	80.80	>82
Photopic 30 Hz flicker amplitude (µV)	Light	3.0	49.5	55.90	>56

Age of affected individual at the time of investigation was 35 years.

### Genetic studies

**Figure 3:**
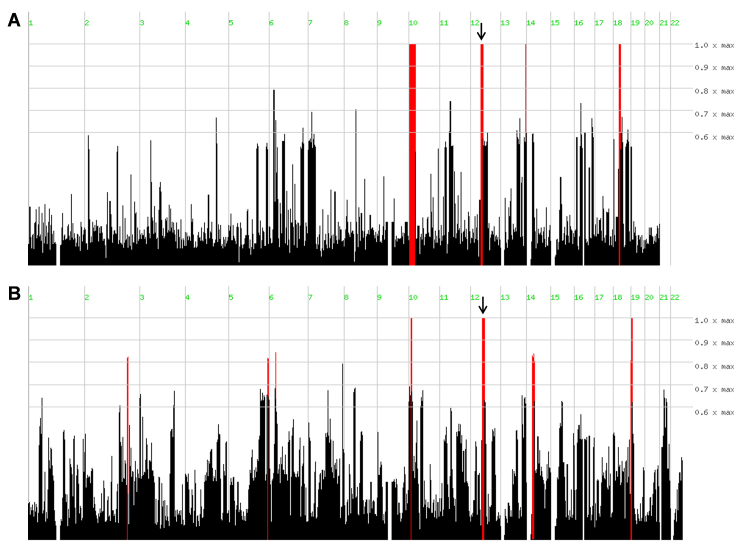
Homozygosity mapping results. **A**: Plot of homozygous regions identified in affected individuals in family A using Homozygosity Mapper analysis. **B**: Plot of homozygous regions identified in affected individuals in family B using Homozygosity Mapper analysis. The red lines indicate homozygous regions shared by affected individuals in each family. The arrows indicate the homozygous regions that harbor *RDH5*.

In family A, three homozygous regions were identified that were shared by the affected persons (Figure 3A). The largest homozygous region spanned 24.5 Mb (hg19: 3.3–27.8 Mb; flanked by SNPs rs4881131 and rs10764698) on chromosome 10. The second and third homozygous regions were 10.5 Mb (hg19: 46.4–56.9 Mb; flanked by rs11183300 and rs7314300) and 8.1 Mb (hg19: 25.9–34.0 Mb; flanked by rs9521585 and rs9555687) in length, and were located on chromosomes 12 and 8, respectively. The second largest region (10.5 Mb) on chromosome 12 harbored the FA-associated gene *RDH5*. *RDH5* sequence analysis identified a novel homozygous 5 bp deletion (c.913_917delGTGCT; p.Val305Hisfs*29) in family A (Figure 1C).

The mutation c.913_917delGTGCT (p.Val305Hisfs*29) segregated in family A (Figure 1A) was consistent with an autosomal recessive inheritance pattern. Both affected individuals carried this mutation in a homozygous state, while both parents and an unaffected brother carried this variant heterozygously. The mutation causes a frameshift in the open reading frame and results in the replacement of the last 14 amino acids of the RDH5 protein by 28 aberrant amino acids. This mutation is predicted to affect part of the transmembrane domain and elongate the cytosolic C-terminal tail. As this deletion is located in the last exon of *RDH5*, nonsense-mediated decay of the mutant mRNA is not predicted.

**Figure 4:**
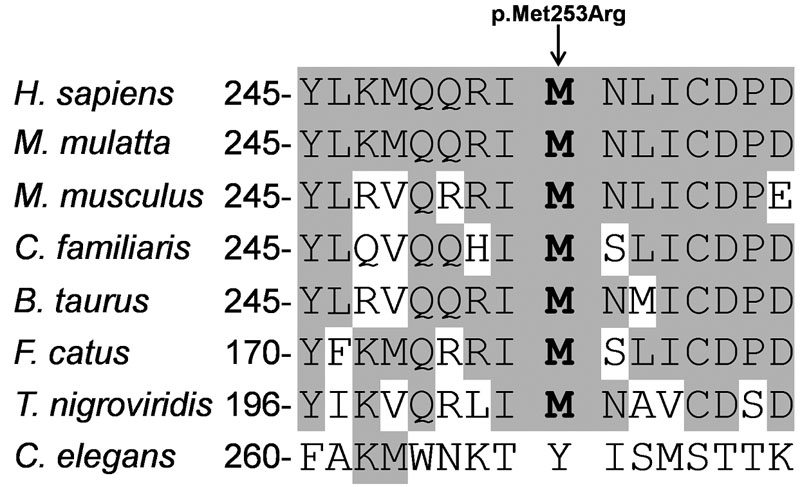
Amino acid conservation of amino acids 245–260 of RDH5 in different species. Gray shading indicates amino acids that are identical to human RDH5 amino acids.

In family B homozygosity mapping revealed an 8.9 Mb (hg19: 52.6–61.5 Mb) homozygous segment (Figure 3B) flanked by SNPs rs1894035 and rs1395538, encompassing the *RDH5* gene. *RDH5* sequence analysis revealed a novel homozygous missense mutation (c.758T>G; p.Met253Arg) in this family. Segregation analysis confirmed that all affected individuals were homozygous for the mutation c.758T>G (p.Met253Arg; Figure 1B), suggesting that this variant may be disease causing. The methionine at position 253 is a highly conserved amino acid residue among different species (Figure 4), and c.758T is an evolutionarily highly conserved nucleotide with a phyloP score of 4.40. SIFT predicted p.Met253Arg to be a deleterious (score: 0.05) mutation, Polyphen classified this mutation as probably damaging (score: 0.992), and Mutation Taster predicted this mutation to be disease causing. Structural analysis showed that there was a difference in charge and size of the wild-type Met253 and the mutant Arg253. The wild-type residue is uncharged, whereas the mutant residue is positively charged. The wild-type residue is buried in the alpha helix and the mutant residue introduces a charge in this buried residue in the core of the protein or protein complex, which can lead to misfolding of the protein. The mutant residue is bigger and probably will not fit in the core of the protein. The hydrophobicities of the wild-type and mutant residue also differ, and therefore, this mutation is likely to cause the loss of hydrophobic interactions in the core of the protein.

Ethnically matched control samples were not tested for these mutations; however, neither variant was found in dbSNP nor in 1000 Genomes.

## Discussion

In this study, we have identified two novel disease-causing mutations in *RDH5* in two unrelated consanguineous families with FA. Both families exhibited typical FA, as was evident from the presence of typical white dots in the midperipheral regions of the retina. In both families, the older patients–IV-1 in family A and IV-7 in family B–had macular degeneration, which might suggest a progressive disease course in these families.

Including our findings, 36 different mutations in *RDH5* associated with FA have been identified to date [7,27-48]. FA patients carrying *RDH5* mutations exhibit high phenotypic variability, ranging from nonprogressive to progressive disease, a variable VA, variation in the density of white dots, and occasionally macular involvement. FA with or without cone dystrophy has also been reported with varying degrees of severity [30,37,48]. A total of 85 FA patients from 68 different families carrying *RDH5* mutations have been identified globally (Table 4, Table 5, and Table 6). These persons were found to exhibit a high variability in phenotype, but the presence of white dots was a common feature. In comparing the different phenotypes and genotypes associated with *RDH5*, it is difficult to establish a valid and clear-cut genotype-phenotype correlation.

**Table 4:** *RDH5* mutations causing fundus albipunctatus.

**Exon/Intron**	**Mutations: Allele 1**	**Mutations: Allele 2**	**Phenotype**	**Families**	**Cases**	**Reference**
Exon 2	c.55A>G (p.Arg19Gly)	wt	DWD	1	1	[48]
Exon 2, 4	c.95delT (p.Phe32Serfs*29)	c.712G>T (p.Gly238Trp)	WD, MA	1	1	[47]
Exon 2, 3	c.98T>A (p.Ile33Asn)	c.469C>T (p.Arg157Trp)	WD	1	1	[39]
Exon 2, 4	c.98T>C (p.Ile33Thr)	c.712G>T (p.Gly238Trp)	DWD, RPED	1	1	[48]
Exon 2	c.103G>A (p.Gly35Ser)	c.103G>A (p.Gly35Ser)	WD, CD, BE	1, 1, 1	2, 1, 1	[30,32,37,46]
Exon 2, 5	c.103G>A (p.Gly35Ser)	c.928delinsGAAG (p.Leu310delinsEV)	WD	1, 2	1, 2	[30,37,41]
Exon 2, 5	c.124C>T (p.Arg42Cys)	c.928delinsGAAG (p.Leu310delinsEV)	WD	1	1	[41]
Exon 2	c.129delT (p.Leu44Trpfs*17)	c.129delT (p.Leu44Trpfs*17)	WD	1	1	[33]
Exon 2, 5	c.211_214dupGTGG (p.Ala72Glyfs*15)	c.801C>G (p.Cys267Trp)	WD	1	1	[33]
Exon 2, 4	c.218C>T (p.Ser73Phe)	c.712G>T (p.Gly238Trp)	WD	1	1	[7]
Intron 2, 4	c.310+1G>A (Splice defect)	c.712G>T (p.Gly238Trp)	DWD	1	1	[48]
Exon 3	c.319G>C (p.Gly107Arg)	c.319G>C (p.Gly107Arg)	WD, MD, SRP	1, 1	2, 1	[35,40]
Exon 3, 5	c.319G>C (p.Gly107Arg)	c.928delinsGAAG (p.Leu310delinsEV)	WD, BE	1	1	[30]

BE, bull’s eye; CD, cone dystrophy; DWD, deep whitish dots; MA, macular atrophy; MD, macular dystrophy; RPED, retinal pigment epithilium degeneration; SRP, sectorial retinitis pigmentosa; WD, white dots.

**Table 5:** *RDH5* mutations causing fundus albipunctatus (cont.).

**Exon/intron**	**Mutations: Allele 1**	**Mutations: Allele 2**	**Phenotype**	**Families**	**Cases**	**Reference**
Exon 3	c.346_347insGCA (p.Gly116_Ile117insSer)	c.346_347insGCA (p.Gly116_Ile117insSer)	DWD, RPED	1	1	[48]
Exon 3, 4	c.346G>C (p.Gly116Arg)	c.710A>C (p.Tyr237Ser)	NWD	1	1	[48]
Exon 3	c.382G>A (p.Asp128Asn)	c.382G>A (p.Asp128Asn)	WD	1	1	[47]
Exon 3, 4	c.382G>A (p.Asp128Asn)	c.712G>T (p.Gly238Trp)	WD	1	1	[43]
Exon 3, 5	c.394G>A (p.Val132Met)	c.839G>A (p.Arg280His)	WD, CD, MD	1, 1, 3	1, 2, 3	[30,37,38,41]
Exon 3, 5	c.416G>T (p.Gly139Val)	c.955T>C (p.*319Argext*33)	DWD, RPED	1	1	[48]
Exon 3	c.470G>A (p.Arg157Gln)	c.470G>A (p.Arg157Gln)	DWD, RPED	1	1	[48]
Exon 3, 4	c.470G>A (p.Arg157Gln)	c.712G>T (p.Gly238Trp)	WD	1	1	[45]
Exon 3	c.490G>T (p.Val164Phe)	c.490G>T (p.Val164Phe)	WD, MA	1	1	[36]
Exon 3, 5	c.530T>G (p.Val177Gly)	c.839G>A (p. Arg280His)	WD	1	1	[29]
Exon 3, 5	c.530T>G (p.Val177Gly)	c.928_930delinsGAAGTT (p.Leu310delinsEV)	WF	1	1	[42]
Exon 4	c.625C>T (p.Arg209*)	c.625C>T (p.Arg209*)	WD	1	1	[47]
Exon 4, 5	c.689_690delinsGG (p.Pro230Arg)	c.928delinsGAAG (p.Leu310delinsEV)	WD	1	1	[44]

CD, cone dystrophy; DWD, deep whitish dots; MA, macular atrophy; MD, macular dystrophy; NWD, no white dots; RPED, retinal pigment epithilium degeneration; WD, white dots; WF, white flecks.

**Table 6:** *RDH5* mutations causing fundus albipunctatus (cont.).

**Exon/intron**	**Mutations: Allele 1**	**Mutations: Allele 2**	**Phenotype**	**Families**	**Cases**	**Reference**
Exon 4	c.712G>T (p.Gly238Trp)	c.712G>T (p.Gly238Trp)	WD, DWD, DWF	1, 1	2, 2, 1	[7,27,48]
Exon 4, 5	c.718dupG (p.Ala240Glyfs*19)	c.841T>C (p.Tyr281His)	WD, BE, MD	1, 1	1, 1	[30,41]
**Exon 5**	**c.758T>G (p.Met253Arg)**	**c.758T>G (p.Met253Arg)**	**WD, MD**	**1**	**5**	**This study**
Exon 5	c.791T>G (p.Val264Gly)	c.791T>G (p.Val264Gly)	WD	1	3	[28]
Exon 5	c.824_825del (p.Arg275Profs*60)	c.824_825del (p.Arg275Profs*60)	DWD, DWF, RPED	1	1	[48]
Exon 5	c.839G>A (p. Arg280His)	c.880G>C (p.Ala294Pro)	WD, MD	1	2	[27]
Exon 5	c.839G>A (p.Arg280His)	c.928delinsGAAG (p.Leu310delinsEV)	WD	1, 1, 2	1, 1, 2	[30,37,40,41]
Exon 5	c.841T>C (p.Tyr281His)	c.928delinsGAAG (p.Leu310delinsEV)	WD, MD	1, 1	1, 1	[34,41]
Exon 5	c.880G>C (p.Ala294Pro)	c.880G>C (p.Ala294Pro)	WD	1	1	[47]
**Exon 5**	c.913_917delGTGCT (p.Val305Hisfs*29)	c.913_917delGTGCT (p.Val305Hisfs*29)	**WD, MD**	**1**	**2**	**This study**
Exon 5	c.928delinsGAAG (p.Leu310delinsEV)	c.928delinsGAAG (p.Leu310delinsEV)	WD, BE, PP	1, 4, 1, 1, 4, 6	1, 4, 2, 1, 6, 6	[28,30,31,37,41]

BE, bull’s eye; DWD, deep whitish dots; DWF, deep whitish flecks; MD, macular dystrophy; PP, photophobia; RPED, retinal pigment epithilium degeneration; WD, white dots. Mutations identified in this study are in bold.

**Figure 5:**
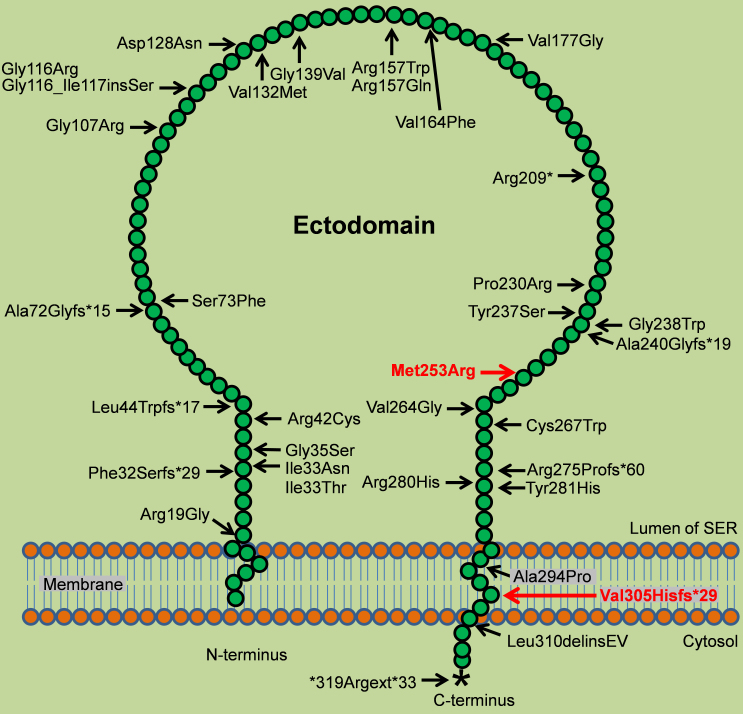
Schematic representation of RDH5 and all mutations thus far published. The membrane-embedded N-terminus consists of 18 amino acids and the ectodomain, present in the lumen of the smooth endoplasmic reticulum (SER), spans amino acids 19–288. A C-terminal membrane-spanning domain encompasses amino acids 289–310, and a small cytosolic tail of eight amino acids resides in the cytosol of retinal pigment epithelium (RPE) cells. Both missense and protein-truncating mutations are distributed across the entire protein. Mutations identified in this study are indicated in red.

RDH5 is a transmembrane enzyme with a membrane-embedded N-terminal domain, a catalytic ectodomain, a C-terminal transmembrane domain, and a cytosolic tail [16]. The topology of retinol dehydrogenases has been controversial as human retinal reductase 1 [49] and mouse retinol dehydrogenase 1 [50] have been reported to have a membrane-embedded N-terminal domain but no C-terminal transmembrane segment, which supports the presence of a cytosolic ectodomain. RDH5 was suggested to have a cytosolic ectodomain without any C-terminal transmembrane domain [50]. However, another retinol dehydrogenase, *cis*-retinol/androgen dehydrogenase 1 (CRAD1), has been described in detail to have a RDH5-like structure with both a luminal ectodomain and cytosolic C-terminal domain, and a similar topology has been suggested for most of the retinol dehydrogenases [51]. The frameshift mutation p.Val305Hisfs*29 identified in family A is located in the C-terminal transmembrane domain, while the missense mutation p.Met253Arg is located in the catalytic ectodomain of RDH5 (Figure 5). As the C-terminal transmembrane region is necessary to retain CRAD1 in the endoplasmic reticulum [51], the *RDH5* mutation p.Val305Hisfs*29 might affect the endoplasmic reticulum localization of RDH5. Moreover, an elongated C-terminal cytosolic tail might also create problems in the proper functioning of RDH5, as the C-terminus is thought to play a role in enzymatic activity and localization of CRAD1 and RDH5 [51].

Structural analysis of RDH5 performed with Project HOPE suggests that the missense mutation p.Met253Arg may cause misfolding of the RDH5 protein because of the loss of hydrophobic interactions in the core of the mutant protein. Misfolding of the mutant protein may cause it to degrade [52-54]. Absence of RDH5 leads to the accumulation of 11-*cis* retinol [20] in the RPE, and a reduction of *11-cis* retinal in the photoreceptors, which in turn might result in the malfunctioning of rod and cone photoreceptor cells.

*RDH5*-associated disease can be prevented with proper genetic counseling of carriers of *RDH5* mutations, and persons with this disease can be treated with 9-*cis*-β-carotene supplementation. *Rdh*^−/−^ mice were successfully treated with 9-*cis* retinal [55], and 9-*cis*-β-carotene was given to FA patients leading to major visual improvements [56]; 9-*cis*-β-carotene is converted to 9-*cis* retinal [57,58], which is more stable than 11-*cis* retinal [59]. The higher stability of opsin bound to 9-*cis* retinal slows down the visual cascade and thus minimizes the toxicity of accumulating by-products in the visual cycle [55,60,61]. In the rod-photoreceptor outer segments 9-*cis* retinol will be converted to all-*trans* retinal during bleaching. This is subsequently reduced to all-*trans* retinol and, in the RPE, all-*trans* retinol is isomerically converted to 9-*cis*, 11-*cis*, and 13-*cis* retinol. A stereospecific enzyme, 9-*cis* retinol dehydrogenase, is reported to be involved in the synthesis of 9-*cis* retinoic acid by oxidizing 9-*cis* retinol [62], and 9-*cis* retinal treatment is suggested to induce the endogenous synthesis of 11-*cis* retinal by its interaction with the retinoid X nuclear receptor [56,59,63].

Based on our and other studies, we estimate that FA contributes to approximately 2% (4/208) of families with retinal dystrophy in Pakistan and a total of 17 patients have been identified with FA [9]. Two FA families have been reported to carry *RLBP1* mutations [9], while two other families with FA have *RDH5* mutations (this study). In the current study, we have identified seven additional FA patients who are candidates for 9-*cis*-β-carotene therapy.

In conclusion, we have identified two novel disease-causing mutations, c.913_917delGTGCT (p.Val305Hisfs*29) and c.758T>G (p.Met253Arg), in two Pakistani families with FA. Our study expands the current mutation spectrum of *RDH5* and contributes to the existing body of knowledge. In addition, this study will help clinicians to improve the diagnosis of FA by differentiating FA from retinitis punctata albescens, providing genetic counseling and prescribing the correct treatment to patients.
